# Phase II pilot study of single-agent etirinotecan pegol (NKTR-102) in bevacizumab-resistant high grade glioma

**DOI:** 10.1007/s11060-015-1795-0

**Published:** 2015-05-03

**Authors:** Seema Nagpal, Cathy Kahn Recht, Sophie Bertrand, Reena Parada Thomas, Abdulrazag Ajlan, Justine Pena, Megan Gershon, Gwen Coffey, Pamela L. Kunz, Gordon Li, Lawrence D. Recht

**Affiliations:** Division of Neuro-Oncology, Department of Neurology, Stanford University, 875 Blake Wilbur Drive CC2221, Stanford, CA 94305 USA; Division of Oncology, Department of Medicine, Stanford University, 875 Blake Wilbur Drive, Stanford, CA 94305 USA; Department of Neurosurgery, Stanford University, 1201 Welch Road MSLS Building p309, Stanford, CA 94305 USA

**Keywords:** Glioma, Glioblastoma, Bevacizumab, Etirinotecan pegol

## Abstract

Patients with recurrence of high-grade glioma (HGG) after bevacizumab (BEV) have an extremely poor prognosis. Etirinotecan pegol (EP) is the first long-acting topoisomerase-I inhibitor designed to concentrate in and provide continuous tumor exposure throughout the entire chemotherapy cycle. Here we report results of a Phase 2, single arm, open-label trial evaluating EP in HGG patients who progressed after BEV. Patients age >18 with histologically proven anaplastic astrocytoma or glioblastoma (GB) who previously received standard chemo-radiation and recurred after BEV were eligible. A predicted life expectancy >6 weeks and KPS ≥ 50 were required. The primary endpoint was PFS at 6-weeks. Secondary endpoint was overall survival from first EP infusion. Response was assessed by RANO criteria. Single agent EP was administered IV every 3 weeks at 145 mg/m2. Patients did not receive BEV while on EP. 20 patients (90 % GB) were enrolled with a median age of 50 and median KPS of 70. Three patients with GB (16.7 % of GB) had partial MRI responses. 6-week PFS was 55 %. Median and 6-month PFS were 2.2 months (95 % CI 1.4–3.4 months) and 11.2 % (95 % CI 1.9–28.9 %) respectively. Median overall survival from first EP infusion was 4.5 months (95 % CI 2.4–5.9). Only one patient had grade 3 toxicity (diarrhea with dehydration) attributable to EP. Hematologic toxicity was mild. Three patients had confirmed partial responses according to RANO criteria. These clinical data combined with a favorable safety profile warrant further clinical investigation of this agent in HGG.

## Introduction

Although there is a desperate need to identify better therapeutic agents for malignant glioma, there are several challenges in clinical design that impede rapid identification and advancement into registration trials. First, the relative infrequency of the disease limits accrual. Second, is the lack of reliable surrogate endpoints [1]. The recent addition of bevacizumab (BEV) to the treatment armamentarium has also confounded assessment. BEV's effect on imaging, its initial efficacy and relatively low side effect profile make it difficult for clinicians to offer trials options that do not include BEV. Therefore, the optimal clinical “space” to screen new agents may be after patients have progression on BEV. Survival in this patient population is usually short, with median survival around 4 months [2–5]. Furthermore, imaging responses are very uncommon; in eight trials, with a total of 192 patients, using non-bevacizumab containing regimens after bevacizumab, there were a total of four partial responses (2 %) [6–13]. From a survival and imaging standpoint, trials can be designed to identify active agents in this space that are active and worthy of further investigation in early lines of treatment.

Topoisomerase- I inhibitors, like irinotecan, have demonstrated some efficacy against HGG [14–17]. Modest efficacy, coupled with the side effect profile, including severe diarrhea and myelosuppression has limited broader use in glioma patients. Etirinotecan pegol (EP) is a next-generation topoisomerase inhibitor formed by steric placement of irinotecan on a four-armed PEG, forming a macromolecular PEG-drug complex that enables prolonged systemic exposure to SN-38, the active metabolite of irinotecan. PEGylated drugs have demonstrated a number of advantages over their precursors: reduced renal clearance, lower propensity for enzymatic breakdown, and extended drug circulation times [18–20]. In theory, extended circulation times may lead to lower overall drug doses, thereby reducing some peak-dose side effects, such as cholenergically mediated diarrhea and myelosuppression. The phase I study of EP confirmed long-acting pharmacokinetics and tolerability. A single dose of EP at 145 mg/m^2^ resulted in the same SN-38 AUC as 350 mg/m^2^ of irinotecan, with a tenfold lower peak SN-38 concentration. The elimination t1/2 of SN-38 for EP was approximately 50 days compared to 12–47 h with irinotecan, demonstrating sustained exposure to the active metabolite [21]. Phase II studies of EP in patients with heavily pre-treated breast cancer and platinum refractory ovarian cancer demonstrated a favorable side effect profile in comparison to a similar schedule of irinotecan, especially with regard to fatigue and bone marrow suppression [22, 23]. Diarrhea does still occur, but tends to be late onset and manageable with a strict diarrhea protocol. Importantly, both of these studies demonstrated encouraging objective response rates (29 and 20 % respectively) in patients who had been exposed to multiple prior agents. Neither study enrolled patients with glioma. However, the ORR in heavily treated patients, prolonged exposure to SN-38, and favorable side effect profile made EP an interesting candidate to study in patients with recurrent high-grade glioma.

In this pilot trial, we studied the tolerability and efficacy of EP in patients with heavily pre-treated, bevacizumab refractory HGG.

## Methods

The study was a prospective, single-arm phase II study conducted at Stanford University (NCT01663012). It was approved by Stanford’s institutional review board and all participants provided written informed consent. Patients were enrolled from August 2012 to May 2013. Adult (>18 years old) patients with recurrent high-grade glioma after the use of bevacizumab were eligible. High grade glioma included WHO grade III and IV tumors with an astrocytic component. Though patients with oligo-astrocytomas were not excluded, no patients with this histology enrolled. All participants had undergone maximally feasible resection (in some cases, this was biopsy alone), standard chemo-radiation or stereotactic radiosurgery concurrent with chemotherapy, had a KPS of at least 50, and had evidence of progression after treatment with bevacizumab. There was no limit on the number of prior lines of therapy. All participants had evidence of adequate bone marrow, renal, and liver function. Patients with pre-existing gastro-intestinal disease leading to acute or chronic diarrhea were excluded.

Patients received treatment with EP mono-therapy at a dose of 145 mg/m^2^ as a 90-min infusion every 21 days. Concurrent treatment with BEV or other cytotoxic agents was not permitted. Treatment with EP continued until time of progression, development of unacceptable side effects, or patient withdrawal from the study. Corticosteroids were allowed at the lowest effective dose to treat symptoms from cerebral edema. Anti-epileptics were used at the discretion of the treating physician. Patients on enzyme inducing anti-epileptics (EIAED) were not expressly excluded, though no patients on EIAEDs were enrolled. Prophylactic anti-emetics were allowed after the initial dose of EP, as needed; pre-medications did not routinely include anti-emetics or atropine. Anti-diarrheal agents were used when diarrhea occurred, but were not permitted as prophylaxis. CTCAE version 4.0 was used to grade toxicity. Due to concern about diarrhea, EP was delayed for any grade of diarrhea experienced within 7 days prior to treatment. Two dose reductions, to 120 and 95 mg/m^2^, were allowed for toxicity.

Complete blood counts and serum chemistry were checked within 7 days prior to each dose of EP. Physical exam, including KPS, and re-assessment of adverse events were performed the day of each infusion. Patients were contacted by phone or email once a week to assess for diarrhea or other adverse events. MRI and physical examination were performed 6 weeks after the first dose of EP. Patients who continued on trial after 6 weeks had MRIs as per standard of care, every 6–10 weeks. Response was measured using RANO criteria and confirmed by a second physician not otherwise participating in patient care. PFS and survivals were calculated from the date of first EP infusion to date of progression or death.

### Trial design and statistics

The primary endpoint was progression free survival at 6 weeks (PFS-6w) as calculated from the first dose of EP. Secondary endpoints were the safety profile of EP in HGG patients, survival from the first dose of EP, and overall survival (OS). This study was powered to compare patients receiving EP to a PFS at 1 month of 5 %. At the time of trial design, there was no clear historical control for post-bevacizumab patients receiving a non-bevacizumab based regimen. Planned enrollment was 20 eligible and evaluable patients, which provided 88 % power to reject a (nominal) PFS-6w rate of 5 % at a one-sided significance level of 10 %, if the true PFS-6w rate was 25 % or better. A mid-enrollment futility assessment was performed after 10 patients reached the 6-week evaluation.

## Results

### Participant characteristics

Twenty patients were enrolled and received their first dose of EP between August 2012 and May 2013. All patients were evaluated for PFS and toxicity. See Table 1 for patient characteristics. The cohort included 18 patients with glioblastoma and 2 patients with anaplastic astrocytoma. MGMT status was available for 14 patients; only 3 patients had promoter-methylated tumor. The median age for participants in this trial was 49.5 (range 20-73) and 8 participants (40 %) were women. The median KPS was 70, with 7 patients (35 %) having a KPS ≤ 60. Nineteen participants had received standard concurrent radiation and temozolomide. One patient had received a concurrent radiosurgery and temozolomide. Median time from diagnosis of HGG to study entry was 1 year and the median number of prior lines of therapy was 3. The median time from diagnosis of HGG to trial enrollment was 12.5 months. The median progression free interval on BEV was 4.8 months. Median time from last BEV dose to first EP dose was 27.5 days. Patients received a median of 3 doses of EP (range 1–22).

**Table 1:** Patient characteristics

Characteristic	N = 20
Median age, years (range)	49.5 (20–73)
Median KPS (0–100) (range)	70 (50–100)
*Histology*	
Primary GB	15 (75 %)
LGG or AA with pathologically confirmed conversion to GB	3 (15 %)
Highest grade anaplastic astrocytoma (III)	2 (10 %)
*Resection*	
Biopsy	7 (35 %)
Sub-total resection	4 (20 %)
Gross total resection	9 (45 %)
Median prior lines of therapy (range)	3 (2–5)
Median time since HGG diagnosis, months (range)	12.5 (3.1–53.0)
Median time since primary diagnosis, months (range)	19.1 (7.0–140.0)

*KPS* Karnofsky performance score, *GB* glioblastoma, *LGG* low-grade glioma, *AA* anaplastic astrocytoma, *HGG* high-grade glioma

### Progression free survival, response, and overall survival

**Fig. 1:**
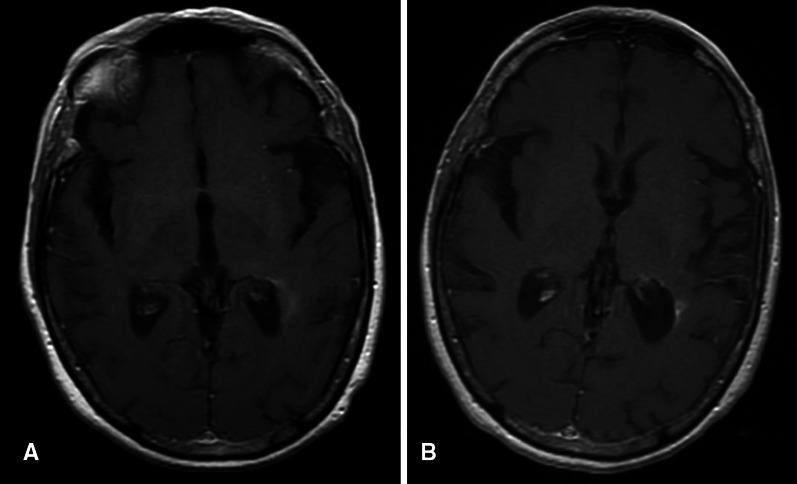
MRI demonstrating a durable response. The patient had a biopsy and treatment of GB anterior and caudal to this lesion. The enhancing area in A appeared and progressed while the patient was receiving BEV, almost a year and a half after first line therapy. The response occurred slowly, over months, while the patient was receiving EP. **a** T1-post contrast at time of progression on BEV, **b** T1-post contrast at approximately 45 weeks on EP

**Fig. 2:**
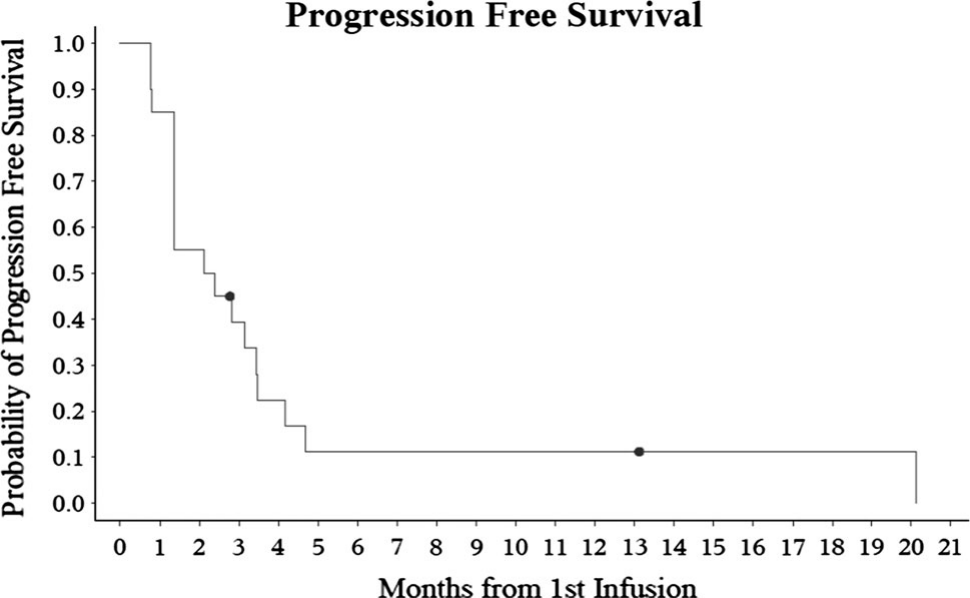
Kaplan–Meier estimate of progression free survival

Partial imaging response (by RANO criteria) was observed in 3 of the 18 GBM patients (16.7 %). See Fig. 1. Five additional GB patients (28 %) had stable disease confirmed at their first and second MRI, bringing total clinical benefit (PR + SD) to 44 %. The 6-week PFS rate was 55 % (95 % CI using exact method, 31.5–76.9 %). The median PFS was 2.2 months (95 % CI 1.4–3.4 months, 2 patients censored) and the 6-month PFS was 11.2 % (95 % CI 1.9–28.9, 2 patients censored). See Fig. 2. The median overall survival from the first infusion of EP was 4.5 months (95 % CI 2.4–5.9, 2 patients censored). One patient is alive, off study, and one patient remains on study. The patient who was unable to follow the diarrhea protocol was censored at the off study date. Patients who were unable to return for follow-up due to clinical deterioration who withdrew from the study for increasing symptoms were considered to have progressive disease. See Table 2 for treatment received following progression on EP.

**Table 2:** Treatment after EP

Regimen	N
No additional anti-tumor treatment/supportive care alone	9
BEV alone	8
BEV + re-irradiation, followed with BEV + BCNU	1
BEV + BCNU	1

*BEV* Bevacizumab

### Toxicity

EP was well tolerated in this heavily pre-treated population. The most common toxicities were grade 1 fatigue 50 %, nausea 60 % and diarrhea 75 %. In the majority of patients with diarrhea, this was a single episode of loose stool. One patient had CTCAE grade 3 diarrhea (this patient was not adherent to the diarrhea supportive care instructions), one patient an asymptomatic grade 3 ALT elevation, and one patient developed grade 3 myelosuppression (pancytopenia). Toxicity led to dose reductions in 2 patients and ultimately, to study discontinuation in the patient who wasn’t able to adhere to supportive care instructions. See Table 3 for grade 3 adverse events attributable to EP. There were no grade 4 or 5 adverse events attributable to EP.

**Table 3:** Grade 3 adverse events attributed to EP. There were no grade 4 or 5 events attributable to EP

Grade 3 adverse events (probably, possibly, or definitely related to EP)	N (%)
Pancytopenia	1 (5)
Diarrhea^a^	2 (10)
Elevated alanine aminotransferase (asymptomatic)	1 (5)
Dehydration^a^	2 (10)
Hypokalemia	1 (5)
Acute kidney injury	1 (5)

^a^Same patient with two separate events of diarrhea and dehydration

## Discussion

Our results suggest EP has single agent activity in this heavily pretreated HGG patients after recurrence on BEV (with a median of three prior regimens). The primary endpoint of median PFS-6 week > 25 % was met; PFS-6 week was 55 %. This short-interval end point, though not validated in the literature, is similar to that of recent trials using short PFS time points in this population [6, 8]. The shorter interval quickly identified progressing patients and allowed rapid assessment of EP’s safety and tolerability in this patient population. In general, our patients were more heavily pre-treated and neurologically affected (35 % patients with a KPS ≤ 60) than patients in comparable trials. The three patients with a KPS of 50 at the time of entry would not likely have been included in other studies. Our study also included 2 patients with AA. While these patients have longer OS from time of diagnosis than their GB counterparts, OS from time of progression on BEV was not well defined at the time this trial was designed. In this study, the diagnosis of AA did not confer benefit; both patients with AA had progressive disease on their first MRI.

The median OS and PFS-6 were 4.5 months and 11.2 % respectively. While the OS and PFS-6 are similar to those seen in recent trials in this population, the imaging response rate of 18 % in GB patients significantly exceeds the 2 % overall response rate seen in prior trials (at 5 % significance, 2 sided). Importantly, two of the responses were highly durable; both in primary GB patients, one who was on study for 20 months and the other who remains on study at 18 months. Neither patient had a significant reduction in tumor volume on their first MRI, but had slow decrease in volume over succeeding months. One patient with SD on the 12 week MRI improved slowly to meet PR criteria over the course of 1 year on study. If prolonged exposure to the topoisomerase inhibitor is required for response, it is possible that use of NKTR-102 in an earlier setting, where patients have potential to receive more cycles of drug, may be more beneficial than in the very heavily pre-treated patient.

Recently, Nowosielski et al. described four different types of tumor progression on BEV and correlated these with survival [3]. Median overall survival after BEV in their cohort was 2.9 months, but patients with progression on BEV that was primarily see on the T2 scans or as a T1-post contrast flare up had median survivals of 4.8 and 4.6 months, respectively. Participants in this study were not stratified by recurrence type. In post hoc analysis, the majority or our patients (14/20) had T2 diffuse or contrast flare-up recurrences. One of the long-term survivors had T2 progression only while the other had increased size in a contrast-enhancing lesion. The third patient in this study with radiographic response was a primary non-responder to BEV.

The 145 mg/m^2^ every 3-week regimen of EP was safe and well tolerated. The major concerns for compounds metabolized to SN-38 are diarrhea and myelosuppression. Only 2 patients (10 %) in this heavily pre-treated cohort, developed grade 3 toxicity related to EP that required clinical intervention. The patient with grade 3 diarrhea was non-adherent to the diarrhea protocol, which calls for the use of loperamide at the first loose stool. This patient was removed from the study 1 month prior to tumor progression, but was censored at the off study date. While loose stool was a frequent complaint, it was easily manageable and did not cause significant dose reduction, delay, or distress in the majority of patients. We did not test for UGT1A mutations in this study, but this could be considered if further studies of EP in glioma are planned.

This study was not powered to demonstrate efficacy of EP over alternative therapies. However, the three PRs noted in this small cohort coupled with the favorable safety profile make EP an attractive candidate for further clinical investigation as a single agent in high-grade gliomas, and potentially, in brain metastases from SN-38 sensitive primary cancers. The combination of EP and BEV is also intriguing. The enhanced permeability and retention effect, proposed by Maeda, postulates that increased vascularity and endothelial permeability in tumors leads to trapping of macromolecules [24]. In theory, administering BEV after EP could amplify this effect by trapping the EP macromolecule as BEV re-normalizes blood vessels. Additionally, if EP requires prolonged exposure to induce response, an earlier setting, such as first recurrence, could increase efficacy.
